# Whole-Exome Sequencing Efficiently Detects Rare Mutations in Autosomal Recessive Nonsyndromic Hearing Loss

**DOI:** 10.1371/journal.pone.0050628

**Published:** 2012-11-30

**Authors:** Oscar Diaz-Horta, Duygu Duman, Joseph Foster, Aslı Sırmacı, Michael Gonzalez, Nejat Mahdieh, Nikou Fotouhi, Mortaza Bonyadi, Filiz Başak Cengiz, Ibis Menendez, Rick H. Ulloa, Yvonne J. K. Edwards, Stephan Züchner, Susan Blanton, Mustafa Tekin

**Affiliations:** 1 John P. Hussman Institute for Human Genomics and the Dr. John T. Macdonald Department of Human Genetics, University of Miami Miller School of Medicine, Miami, Florida, United States of America; 2 Division of Pediatric Genetics, Ankara University School of Medicine, Ankara, Turkey; 3 Growth and Development Research Center, Tehran University of Medical Sciences, Tehran, Iran; 4 Faculty of Natural Sciences, Center of Excellence for Biodiversity, University of Tabriz, Tabriz, Iran; Innsbruck Medical University, Austria

## Abstract

Identification of the pathogenic mutations underlying autosomal recessive nonsyndromic hearing loss (ARNSHL) is difficult, since causative mutations in 39 different genes have so far been reported. After excluding mutations in the most common ARNSHL gene, *GJB2*, via Sanger sequencing, we performed whole-exome sequencing (WES) in 30 individuals from 20 unrelated multiplex consanguineous families with ARNSHL. Agilent SureSelect Human All Exon 50 Mb kits and an Illumina Hiseq2000 instrument were used. An average of 93%, 84% and 73% of bases were covered to 1X, 10X and 20X within the ARNSHL-related coding RefSeq exons, respectively. Uncovered regions with WES included those that are not targeted by the exome capture kit and regions with high GC content. Twelve homozygous mutations in known deafness genes, of which eight are novel, were identified in 12 families: *MYO15A*-p.Q1425X, -p.S1481P, -p.A1551D; *LOXHD1*-p.R1494X, -p.E955X; *GIPC3*-p.H170N; *ILDR1*-p.Q274X; *MYO7A*-p.G2163S; *TECTA*-p.Y1737C; *TMC1*-p.S530X; *TMPRSS3*-p.F13Lfs*10; *TRIOBP*-p.R785Sfs*50. Each mutation was within a homozygous run documented via WES. Sanger sequencing confirmed co-segregation of the mutation with deafness in each family. Four rare heterozygous variants, predicted to be pathogenic, in known deafness genes were detected in 12 families where homozygous causative variants were already identified. Six heterozygous variants that had similar characteristics to those abovementioned variants were present in 15 ethnically-matched individuals with normal hearing. Our results show that rare causative mutations in known ARNSHL genes can be reliably identified via WES. The excess of heterozygous variants should be considered during search for causative mutations in ARNSHL genes, especially in small-sized families.

## Introduction

Hearing loss is one of the most common sensory disorders in humans. Genetic factors account for more than 50% of cases with congenital hearing loss where the majority exhibit autosomal recessive inheritance [1], [2]. Identification of the responsible gene/mutation in affected families is difficult since there are 39 different genes involved in ARNSHL [3] (http://hereditaryhearingloss.org/). Except for mutations in *GJB2*, which are the cause in up to 50% of families with ARNSHL in some populations, most deafness mutations are private and are seen in only a single or few families [4]. Approximately 85% of disease-related mutations in Mendelian disorders have been found in the protein-coding regions (exons and splice sites), although this portion constitutes only approximately 1% of the human genome [5]. Many of ARNSHL genes consist of long and/or many exons making conventional genetic methods to screen for mutations extremely expensive and time-consuming (e.g. Sanger sequencing alone or in combination with genome wide SNP genotyping).Whole-exome sequencing (WES) has recently been introduced as an alternative approach to more traditional methods [6]. WES allows for a targeted enrichment and resequencing of nearly all exons of protein-coding genes. Exome sequences are analyzed to identify genetic variation at a base-pair resolution and survey the protein coding portion of the human genome. Accordingly, WES using next generation technologies provides a transformational approach for identifying causative mutations in Mendelian disorders. Different targeted genomic capture methods and massive parallel sequencing have been successfully applied to screen mutations in hereditary deafness in relatively small sets of families [7]–[9]. In the present work, we applied WES to ARNSHL in a set of 20 multiplex and consanguineous families. The systematic use of WES shows that this method is an effective mean to identify causative mutations in affected families.

## Materials and Methods

### Ethics Statement

This study was approved by the University of Miami Institutional Review Board (USA), Ankara University Medical School Ethics Committee (Turkey), and Growth and Development Research Ethics Committee (Iran). All participants provided written informed consent prior to enrollment. Written informed consent was obtained from the next of kin on the behalf of the minors/children participants involved in this study.

### Subjects

Twenty families were included in this study based on the presence of both parental consanguinity and at least two members with hearing loss (Figure S1). Seventeen families were from Turkey and three were from Iran. Diagnosis of sensorineural hearing loss was established via standard audiometry in a sound-proofed room according to current clinical standards [10]. Clinical evaluation of all affected individuals by a geneticist and an ENT surgeon included a thorough physical examination and otoscopy. A high resolution CT scan of the temporal bone was obtained in one affected person in each family to look for inner ear anomalies. DNA was extracted from peripheral leukocytes of each member of the family via a phenol chloroform method. Samples were prescreened for mutations in *GJB2* (MIM 121011) via Sanger sequencing; none were identified in any of the 20 families. Either one or two affected members of each family underwent WES based on the availability of sufficient DNA.

### Whole-Exome Sequencing

The SureSelect Human All Exon 50 Mb kit (Agilent) was used for in-solution enrichment of coding exons and flanking intronic sequences following the manufacturer’s standard protocol. Adapter sequences for the Illumina Hiseq2000 were ligated and the enriched DNA samples were subjected to standard sample preparation for the Hiseq2000 instrument (Illumina). The Illumina CASAVA v1.8 pipeline was used to produce 99 bp sequence reads. BWA [11] was used to align sequence reads to the human reference genome (hg19) and variants were called using the GATK software package [12], [13]. All variants were submitted to SeattleSeq134 for further characterization. Coverage analysis was performed on coding exons +/−2 bp of cDNA transcripts listed in the Human Gene Mutation Database (https://portal.biobase-international.com) of known ARNSHL genes, which is comprised of 115,447 bps and 782 exons. Positions of heterozygous and homozygous variants were used to determine the location and size of the homozygous blocks containing the mutations.

### Sanger Sequencing

Candidate variants observed via WES were confirmed using conventional capillary sequencing. The primers were designed using Primer3, v. 0.4.0 (http://frodo.wi.mit.edu/). PCR reactions included 10–40 ng of genomic DNA with Taq DNA polymerase (Roche). Corresponding DNA fragments were amplified using a touch down protocol. PCR products were visualized on agarose gels, cleaned over Sephadex columns or with NucleoFast 96 PCR plates (Clontech) in accordance with the manufacturers’ protocols. Sequence analysis was performed with the ABI PRISM Big Dye Terminator Cycle Sequencing V3.1 Ready Reaction Kit and the ABI PRISM 3730 DNA Analyzer (Applied Biosystems). Sequence traces were analyzed using the Sequencher 4.7 program (Gene Codes Corporation).

### *In silico* Analysis of Identified Variants

The program Conseq was used to calculate conservation scores for the amino acid residue affected by the missense variants [14]. Scores ranged from 1–9, where a score of 9 represented a highly conserved residue. PolyPhen-2 [15] and SIFT [16] were used to predict the effect of reported missense changes on protein function.

## Results

### Exome Sequencing

**Figure 1:**
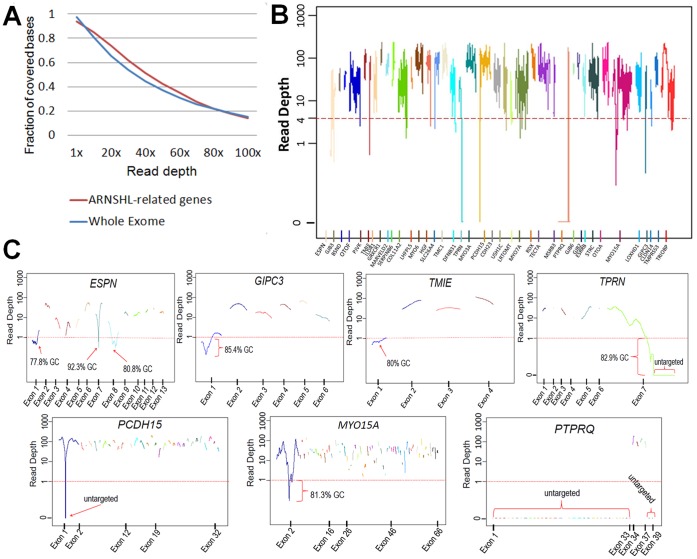
A) The exome coverage of the 39 ARNSHL genes. The plots are the average of the fraction of covered bases (Y-axis) and the read depth (X-axis) in the 20 families included in the study. B) Average sequence coverage (base 10 logarithmic scale) of ARNSHL genes. Exome sequencing results in a range of sequence coverage depth. C) Detailed uncovered segments of ARNSHL genes. Untargeted and high GC content sequences are indicated.

On average, each exome in the 20 families had 97%, 81% and 66% of mappable bases of the Gencode defined exome represented by coverage of at least 1, 10 and 20 reads, respectively. Considering only the 39 selected ARNSHL-related genes the percentage of mappable bases was 93%, 84% and 73%, respectively (Fig. 1A). An average depth of 53.4 reads was achieved for all known ARNSHL genes. The average quality of single nucleotide variations and insertions or deletions (INDELs) was 798 and 1014, respectively. Uncovered regions (read depth <1) within the known ARNSHL genes were mostly limited to small segments (less than 200 bp) in seven genes (Fig. 1B and 1C), except for *PTPRQ* of which only four out of 39 exons were targeted. Poor coverage was associated with untargeted regions of the capture kit as well as with segments having an excessively high GC content (Fig. 1C).

### Identification of ARNSHL Mutations

An average of 90,237 single nucleotide variations and 8,059 INDELs per sample were found prior to applying any filter. We utilized the Genomes Management Application (GEMapp), University of Miami Miller School of Medicine (https://secureforms.med.miami.edu/hihg/gem-app) for filtering of variants. We filtered the variants according to the inheritance model (autosomal recessive with both homozygous and compound heterozygous), the variant function class including missense, nonsense, splice sites, in-frame INDELs and frame-shift INDELs and for the following genes: *BSND, CDH23, CLDN14, COL11A2, DFNB31, ESPN, ESRRB, GIPC3, GJB2, GJB3, GJB6, GRXCR1, HGF, ILDR1, LHFPL5, LOXHD1, LRTOMT, MARVELD2, MSRB3, MYO15A, MYO3A, MYO6, MYO7A, OTOA, OTOF, PCDH15, PJVK, PTPRQ, RDX, SERPINB6, SLC26A4, STRC, TECTA, TMC1, TMIE, TMPRSS3, TPRN, TRIOBP,* and *USH1C*. We searched for variants that were not listed in dbSNP137, NHLBI (http://evs.gs.washington.edu/EVS/) or that had a minor allele frequency of less than 1% in these databases. Variants were also filtered for presence in less than five samples in our internal database that includes 1010 exomes, including 69 ethnicity matched unrelated samples. We did not use a filter for read depth and included even variants with 1×coverage because we were searching for homozygous variants. This simple filtering strategy rapidly extracted only 1 homozygous variant in one of the searched ARNSHL genes in 12 families. Subsequent Sanger sequencing confirmed co-segregation of the mutations with the phenotype (Table 1 and Figure S1). For the five missense mutations detected, estimated conservation and pathogenicity scores (Table 1) suggested that single amino acid changes were likely to be highly damaging to protein function. Moreover, all single amino acid changes were located within conserved domains of the protein (Table 1). In the affected members of the remaining families, we looked for homozygous haplotypes flanking *ESPN, GIPC3, TMIE, TPRN, PCDH15, MYO15A,* and *PTPRQ*, which were not fully covered by WES. This analysis did not show homozygous runs in any family suggesting that autozygous mutations in those genes are unlikely. As shown in Table S1, all identified mutations were within homozygous runs. Our work searching for novel deafness genes in other autozygous areas in the remaining families is ongoing.

**Table 1:** Mutations identified in the ARNSHL-related genes.

FamilyID	# exomessequenced	Origin	Gene	cDNA change^1^	Amino acidchange^1^	ConseqScore	PolyPhen-2	SIFT	Affected ProteinDomain	Reference
1	2	Turkey	*TRIOBP*	c.2355_2356delAG	p.R785Sfs*50	n/a	n/a	n/a	n/a	Novel
2	2	Turkey	*TMC1*	c.1589_1590delCT	p.S530X	n/a	n/a	n/a	n/a	[18]
3	1	Turkey	*LOXHD1*	c.4480C>T	p.R1494X	n/a	n/a	n/a	n/a	Novel
4	2	Turkey	*TMPRSS3*	c.36dupC	p.F13Lfs*10	n/a	n/a	n/a	n/a	Novel
5	1	Turkey	*MYO15A*	c.4441T>C	p.S1481P	4	1.000	0.01	Myosin MotorDomain	[19]
6	2	Turkey	*MYO15A*	c.4652C>A	p.A1551D	7	1.000	0.0	Myosin MotorDomain	Novel
7	1	Turkey	*MYO15A*	c.4273C>T	p.Q1425X	n/a	n/a	n/a	n/a	Novel
8	1	Turkey	*LOXHD1*	c.2863G>T	p.E955X	n/a	n/a	n/a	n/a	Novel
9	2	Turkey	*GIPC3*	c.508C>A	p.H170N	5	0.991	0.04	PDZ Domain	[20]
10	1	Iran	*ILDR1*	c.820C>T	p.Q274X	n/a	n/a	n/a	n/a	Novel
11	1	Iran	*MYO7A*	c.6487G>A	p.G2163S	9	1.000	0.0	FERM CentralDomain	[21]
12	1	Iran	*TECTA*	c.5210A>G	p.Y1737C	7	0.999	0.0	C8 domain	Novel

1 According to cDNA and protein sequences used for each gene in the HGMD.

During the analysis we also noticed an unexpectedly high frequency (four variants in 12 families) of heterozygous variants in known deafness genes in families where homozygous causative mutations were already identified (Table S2). Variants were selected based on a <1% frequency in the dbSNP137 database, high pathogenicity scores calculated by Polyphen-2 and SIFT and having at least 20X read depth. Using our internal WES database, the frequency of heterozygous variants (same criteria applied) in 39 ARNSHL genes were also determined in hearing individuals (n = 15) matched for ethnicity (Table S2). There were six heterozygous variants in 15 normal hearing individuals.

## Discussion

WES allows for screening of mutations in a large number of genes at the same time and is cost effective compared to other genetic testing options when the number of genes is large. This is particularly true when the condition under study is extremely heterogeneous, as with ARNSHL. Although very small regions are not adequately covered, the present study indicates that our current WES protocol can reliably test most of the deafness genes. Limitations inherent in the processes of exome enrichment and amplification are evident mostly because of a high GC content (Fig. 1C) and because the SureSelect Human All Exon 50 Mb kit does not capture all the nucleotides of clinical interest. Most exons of *PTPRQ* in particular are not targeted by this exome capture kit. To overcome this, Sanger sequencing of the poorly targeted genes can be performed. If the study population includes multiplex and/or consanguineous families, such as those studied in this report, co-segregation of a haplotype flanking a known gene can be easily assessed with the WES data. We used the latter approach for the assessment of homozygosity around the uncovered regions. We previously showed that compound heterozygous mutations are detected in less than 5% of consanguineous families with deafness [3]. Thus, it is unlikely that the families in which we did not identify a homozygous haplotype flanking the poorly covered regions harbor compound heterozygous mutations in these genes.

Our dataset allows us to perform an additional analysis that is relevant for the clinical usage of next-generation sequencing: since we have already found the cause of deafness in our 12 families, we can then look for other variants in the same genes that have the potential to be pathogenic. As shown in Table S2, four out of 12 families with hearing loss caused by homozygous mutations in known deafness genes also carried heterozygous variants that could potentially cause hearing loss in the homozygous state. Our subsequent analysis in 15 ethnically-matched hearing individuals revealed six similar variants, suggesting that this observation is not limited to families with deafness. Although these variants are predicted to damage the corresponding protein function, it is unlikely for most of them that they cause prelingual deafness because their frequencies are too high in both hearing and deaf families. They might have the potential to modify the phenotype or contribute to late onset hearing loss. It is important to recognize that there is an excess of heterozygous, rare, and predicted to be pathogenic variants in known deafness genes. This is especially relevant for simplex cases or small-sized families where co-segregation of the identified variant cannot be investigated in a large pedigree.

Our study corroborates the extreme heterogeneity of ARNSHL. Within the twenty family exome set, there was not a single recurrent mutation causing deafness. In addition, eight out of the 12 mutations in known deafness genes are novel (Table 1). These data also indicate the great potential that WES provides for the discovery of novel deafness genes. We can then concentrate on the remaining eight families to identify mutations in novel genes for ARNSHL.

As discussed in a recent review by Shearer and Smith, targeted genome enrichment (e.g. subsets of deafness-related genes) followed by massively parallel sequencing constitute an efficient strategy for deafness gene mutation screening [17]. Two independent studies screening more than 50 known deafness genes reported the identification of causative mutations in 5 out of 6 and 6 out of 11 families, respectively [8], [9]. This approach has several advantages over WES including: a higher coverage (the reads are not “diluted” over more than 20,000 genes), and a significant lowering of costs (e.g. the lower the capture size the higher number of samples per HiSeq lane can be processed). However, custom enrichment has limitations, especially when considering that a minimum of 34% of deafness genes have yet to be discovered [17].Thus, if the causative mutation resides in novel genes, additional experiments will be required. In this sense, custom capture kits for deafness genes should be subject to periodical updates that may negatively influence their price. WES allows performing post *in silico* analysis as the stored data comprises the entire coding sequence of the genome.

In summary, we have successfully applied WES for mutation screening within ARNSHL-related genes. The high percentage of unsolved mutations in those genes indicates the great potential of novel gene discovery in consanguineous families utilizing whole exome enrichment methods and next generation sequencing.

## Supporting Information

Figure S1 **Pedigrees and electropherograms showing segregating mutations in known deafness genes.** The levels of consanguinity are indicated; −/−: homozygous mutant; +/−: heterozygous; +/+: homozygous wild type.
(PDF)

Table S1 **Size and location of the homozygous blocks containing the causative mutations in the studied families.** Asterisks indicate families in which homozygous blocks were calculated by overlapping sequence data from two individuals.
(PDF)

Table S2 **Heterozygous mutations in known deafness genes in affected families and hearing individuals. HGMD: Human Gene Mutation Database.** ^1^Minor allele frequency is from dbSNP137 database accessed on 10/17/2012. ^2^Internal database allele frequency does not include the family on this table with a given variant.
(PDF)
