# Correction: Whole-Exome Sequencing Efficiently Detects Rare Mutations in Autosomal Recessive Nonsyndromic Hearing Loss

**DOI:** 10.1371/annotation/0668e8de-97ae-4a04-ac61-8afa9c79bbdd

**Published:** 2013-05-17

**Authors:** Oscar Diaz-Horta, Duygu Duman, Joseph Foster, Aslı Sırmacı, Michael Gonzalez, Nejat Mahdieh, Nikou Fotouhi, Mortaza Bonyadi, Filiz Başak Cengiz, Ibis Menendez, Rick H. Ulloa, Yvonne J. K. Edwards, Stephan Züchner, Susan Blanton, Mustafa Tekin

The affiliation for the 6th author is missing. Nejat Mahdieh is affiliated with #3. Affiliation #3 was incomplete in the PDF document. Affiliation number 3 is: Growth and Development Research Center, Tehran University of Medical Sciences, Tehran and Faculty of Medicine, Ilam University of Medical Sciences, Ilam, Iran

