# Supplementary material for: Whole-Exome Sequencing Efficiently Detects Rare Mutations in Autosomal Recessive Nonsyndromic Hearing Loss
Source: PLoS One. 2012 Nov 30;7(11):e50628. doi: 10.1371/journal.pone.0050628 (PMC3511533; doi:10.1371/journal.pone.0050628)
Supplement: Figure S1 — Pedigrees and electropherograms showing segregating mutations in known deafness genes. The levels of consanguinity are indicated; −/−: homozygous mutant; +/−: heterozygous; +/+: homozygous wild type. (PDF) [file pone.0050628.s001.pdf]

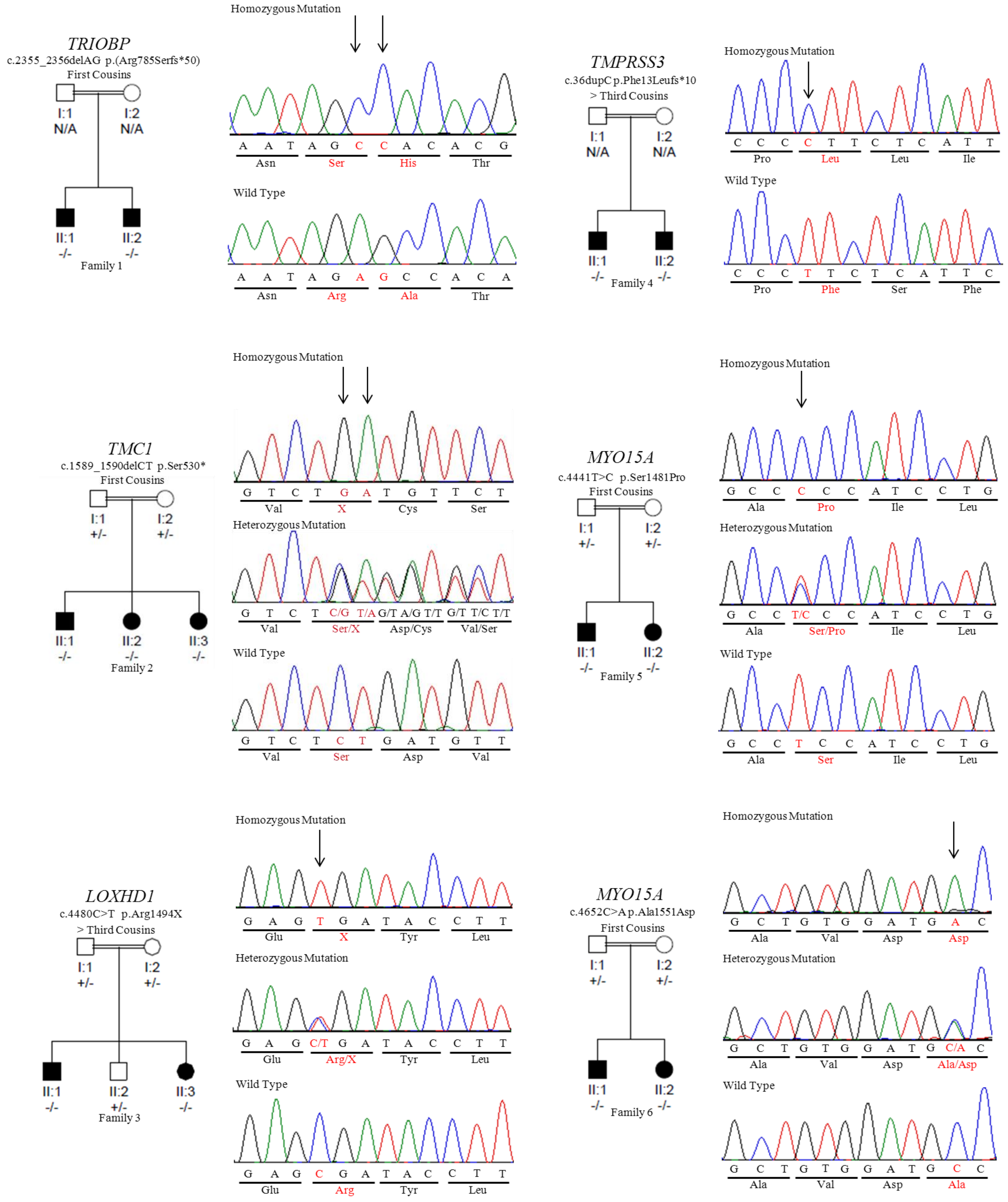

TRIOBP
c.2355_2356delAG p.(Arg785Serfs*50)
First Cousins
I:1 N/A
I:2 N/A
II:1 -/-
II:2 -/-
Family 1
Homozygous Mutation
A A T A G C C A C A C G
Asn Ser His Thr
Wild Type
A A T A G A G C C A C A
Asn Arg Ala Thr
TMPRSS3
c.36dupC p.Phe13Leufs*10
> Third Cousins
I:1 N/A
I:2 N/A
II:1 -/-
II:2 -/-
Family 4
Homozygous Mutation
C C C C T T C T C A T T
Pro Leu Leu Ile
Wild Type
C C C T T C T C A T T C
Pro Phe Ser Phe
TMC1
c.1589_1590delCT p.Ser530*
First Cousins
I:1 +/-
I:2 +/-
II:1 -/-
II:2 -/-
II:3 -/-
Family 2
Homozygous Mutation
G T C T G A T G T T C T
Val X Cys Ser
Heterozygous Mutation
G T C T C/G T/A G/T A/G T/T G/T T/C T/T
Val Ser/X Asp/Cys Val/Ser
Wild Type
G T C T C T G A T G T T
Val Ser Asp Val
MYO15A
c.4441T>C p.Ser1481Pro
First Cousins
I:1 +/-
I:2 +/-
II:1 -/-
II:2 -/-
Family 5
Homozygous Mutation
G C C C C C A T C C T G
Ala Pro Ile Leu
Heterozygous Mutation
G C C T/C C C A T C C T G
Ala Ser/Pro Ile Leu
Wild Type
G C C T C C A T C C T G
Ala Ser Ile Leu
LOXHD1
c.4480C>T p.Arg1494X
> Third Cousins
I:1 +/-
I:2 +/-
II:1 -/-
II:2 +/-
II:3 -/-
Family 3
Homozygous Mutation
G A G T G A T A C C T T
Glu X Tyr Leu
Heterozygous Mutation
G A G C/T G A T A C C T T
Glu Arg/X Tyr Leu
Wild Type
G A G C G A T A C C T T
Glu Arg Tyr Leu
MYO15A
c.4652C>A p.Ala1551Asp
First Cousins
I:1 +/-
I:2 +/-
II:1 -/-
II:2 -/-
Family 6
Homozygous Mutation
G C T G T G G A T G A C
Ala Val Asp Asp
Heterozygous Mutation
G C T G T G G A T G C/A C
Ala Val Asp Ala/Asp
Wild Type
G C T G T G G A T G C C
Ala Val Asp Ala

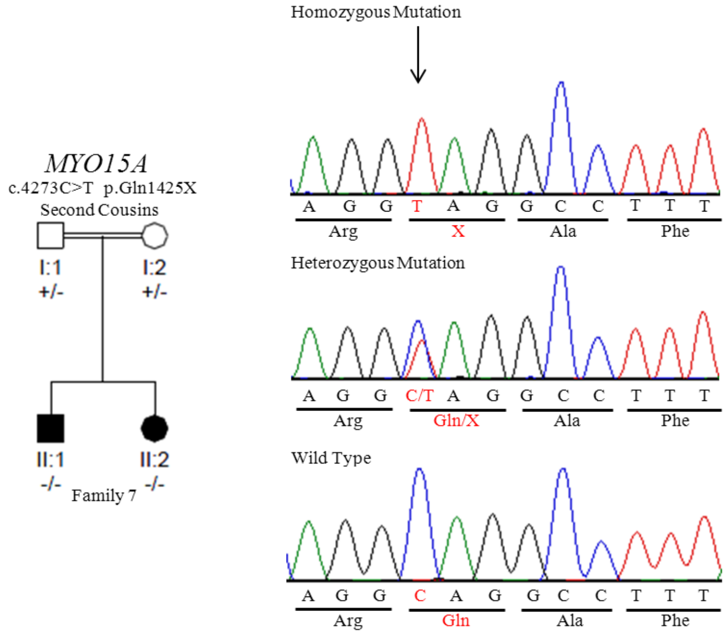
Homozygous Mutation
A G G T A G G C C T T T
Arg X Ala Phe
Heterozygous Mutation
A G G C/T A G G C C T T T
Arg Gln/X Ala Phe
Wild Type
A G G C A G G C C T T T
Arg Gln Ala Phe
MYO15A
c.4273C>T p.Gln1425X
Second Cousins
I:1
+/-
I:2
+/-
II:1
-/-
II:2
-/-
Family 7

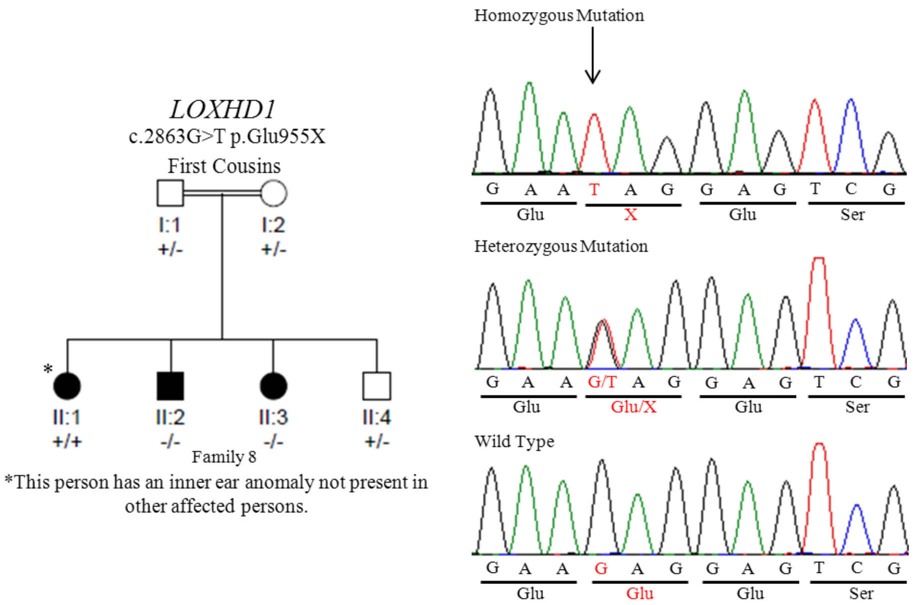
Homozygous Mutation
G A A T A G G A G T C G
Glu X Glu Ser
Heterozygous Mutation
G A A G/T A G G A G T C G
Glu Glu/X Glu Ser
Wild Type
G A A G A G G A G T C G
Glu Glu Glu Ser
LOXHD1
c.2863G>T p.Glu955X
First Cousins
I:1
+/-
I:2
+/-
*
II:1
+/+
II:2
-/-
II:3
-/-
II:4
+/-
Family 8
*This person has an inner ear anomaly not present in other affected persons.

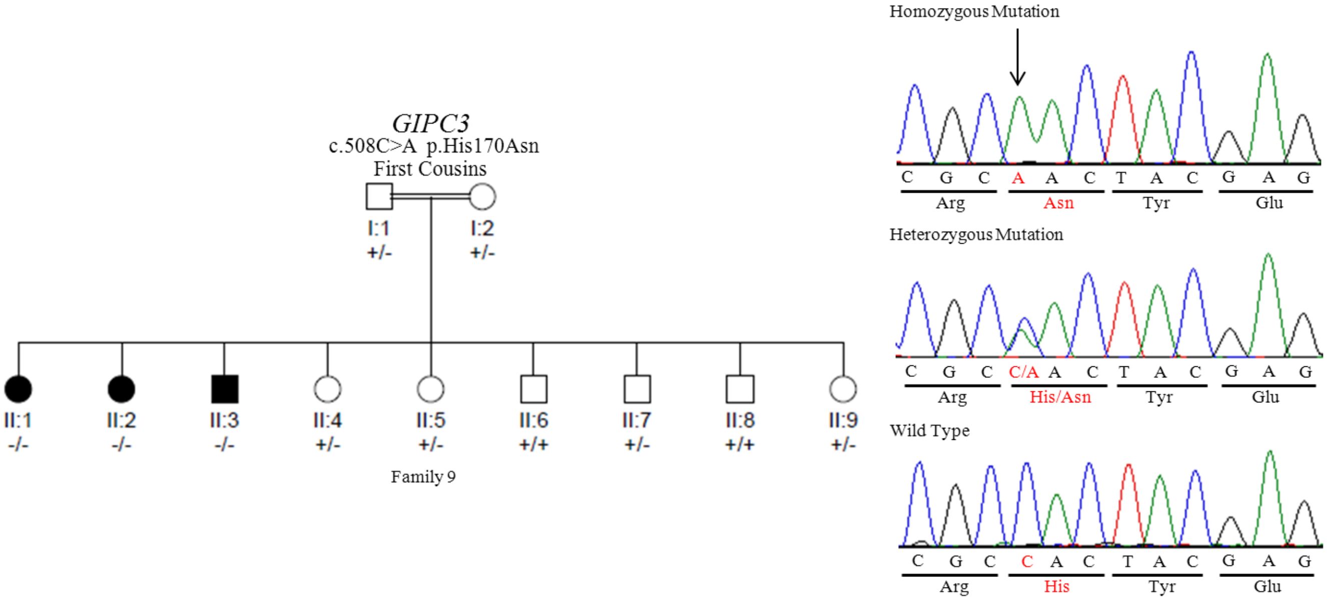
Homozygous Mutation
C G C A A C T A C G A G
Arg Asn Tyr Glu
Heterozygous Mutation
C G C C/A A C T A C G A G
Arg His/Asn Tyr Glu
Wild Type
C G C C A C T A C G A G
Arg His Tyr Glu
GIPC3
c.508C>A p.His170Asn
First Cousins
I:1
+/-
I:2
+/-
II:1
-/-
II:2
-/-
II:3
-/-
II:4
+/-
II:5
+/-
II:6
+/+
II:7
+/-
II:8
+/+
II:9
+/-
Family 9

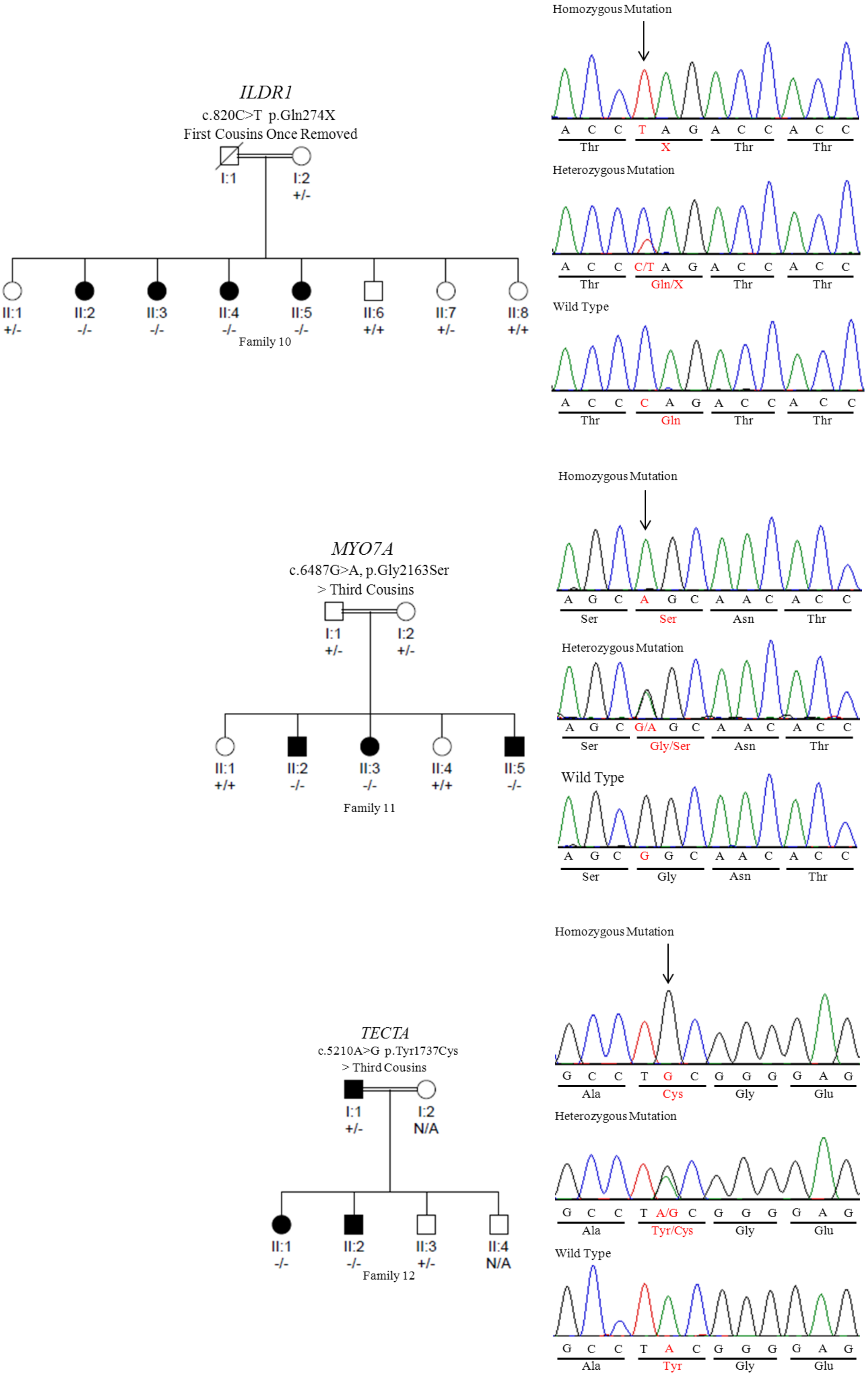
ILDR1
c.820C>T p.Gln274X
First Cousins Once Removed
I:1
I:2
+/-
II:1 +/-
II:2 -/-
II:3 -/-
II:4 -/-
II:5 -/-
II:6 +/+
II:7 +/-
II:8 +/+
Family 10
Homozygous Mutation
A C C T A G A C C A C C
Thr X Thr Thr
Heterozygous Mutation
A C C C/T A G A C C A C C
Thr Gln/X Thr Thr
Wild Type
A C C C A G A C C A C C
Thr Gln Thr Thr
MYO7A
c.6487G>A, p.Gly2163Ser
> Third Cousins
I:1 +/-
I:2 +/-
II:1 +/+
II:2 -/-
II:3 -/-
II:4 +/+
II:5 -/-
Family 11
Homozygous Mutation
A G C A G C A A C A C C
Ser Ser Asn Thr
Heterozygous Mutation
A G C G/A G C A A C A C C
Ser Gly/Ser Asn Thr
Wild Type
A G C G G C A A C A C C
Ser Gly Asn Thr
TECTA
c.5210A>G p.Tyr1737Cys
> Third Cousins
I:1 +/-
I:2 N/A
II:1 -/-
II:2 -/-
II:3 +/-
II:4 N/A
Family 12
Homozygous Mutation
G C C T G C G G G G A G
Ala Cys Gly Glu
Heterozygous Mutation
G C C T A/G C G G G G A G
Ala Tyr/Cys Gly Glu
Wild Type
G C C T A C G G G G A G
Ala Tyr Gly Glu

Article title: Whole-exome sequencing efficiently detects rare mutations in autosomal recessive nonsyndromic hearing loss

Journal name: Human Genetics

Author names: Oscar Diaz-Horta[1], Duygu Duman[2], Joseph Foster II[1], Aslı Sırmacı[1], Michael Gonzalez[1], Nejat Mahdieh[3], Nikou Fotouhi[4], Mortaza Bonyadi[4], Filiz Başak Cengiz[2], Ibis Menendez[1], Rick H. Ulloa[1], Yvonne J.K. Edwards[1], Stephan Züchner[1], Susan Blanton[1], Mustafa Tekin[1]

Affiliations:

1) John P. Hussman Institute for Human Genomics and the Dr. John T. Macdonald Department of Human Genetics, University of Miami Miller School of Medicine, Miami, USA

2) Division of Pediatric Genetics, Ankara University School of Medicine, Ankara, Turkey

3) Growth and Development Research Center, Tehran University of Medical Sciences, Tehran, Iran

4) Faculty of Natural Sciences, Center of Excellence for Biodiversity, University of Tabriz, Tabriz, Iran

Email address of the corresponding author: mtekin@med.miami.edu
