# Supplementary material for: Whole-Exome Sequencing Efficiently Detects Rare Mutations in Autosomal Recessive Nonsyndromic Hearing Loss
Source: PLoS One. 2012 Nov 30;7(11):e50628. doi: 10.1371/journal.pone.0050628 (PMC3511533; doi:10.1371/journal.pone.0050628)
Supplement: Table S1 — Size and location of the homozygous blocks containing the causative mutations in the studied families. Asterisks indicate families in which homozygous blocks were calculated by overlapping sequence data from two individuals. (PDF) [file pone.0050628.s002.pdf]

Family #	Gene	Size of Homozygous block (Mb)	Homozygous blocks (rectangles) and gene localization (triangles) in chromosomes.
1*	*TRIOBP*	2.74	
2*	*TMC1*	14.02	
3	*LOXHD1*	10.85	
4*	*TMPRSS3*	4.05	
5	*MYO15A*	1.14	
6*	*MYO15A*	3.07	
7	*MYO15A*	2.55	
8	*LOXHD1*	58.13	
9*	*GIPC3*	3.43	
10	*ILDR1*	4.66	
11	*MYO7A*	6.86	
12	*TECTA*	9.66	

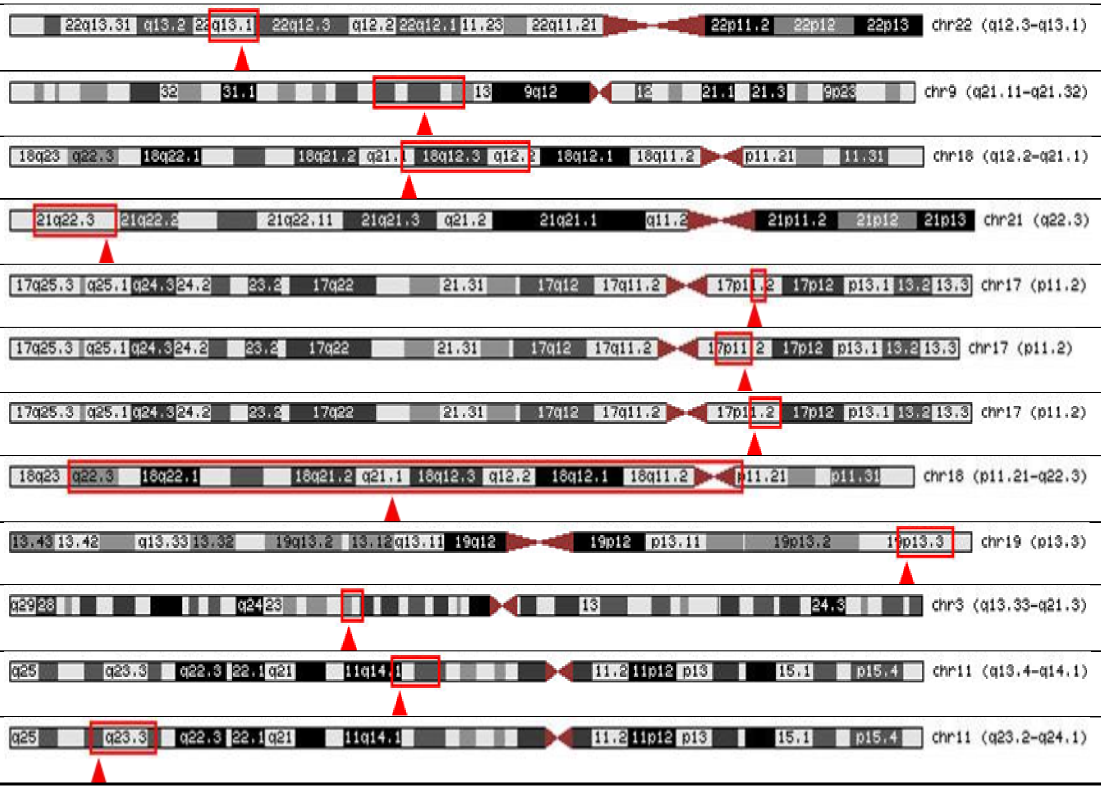

Article title: Whole-exome sequencing efficiently detects rare mutations in autosomal recessive nonsyndromic hearing loss

Journal name: Human Genetics

Author names: Oscar Diaz-Horta[1], Duygu Duman[2], Joseph Foster II[1], Aslı Sırmacı[1], Michael Gonzalez[1], Nejat Mahdieh[3], Nikou Fotouhi[4], Mortaza Bonyadi[4], Filiz Başak Cengiz[2], Ibis Menendez[1], Rick H. Ulloa[1], Yvonne J.K. Edwards[1], Stephan Züchner[1], Susan Blanton[1], Mustafa Tekin[1]

Affiliations:

1) John P. Hussman Institute for Human Genomics and the Dr. John T. Macdonald Department of Human Genetics, University of Miami Miller School of Medicine, Miami, USA

2) Division of Pediatric Genetics, Ankara University School of Medicine, Ankara, Turkey

3) Growth and Development Research Center, Tehran University of Medical Sciences, Tehran, Iran

4) Faculty of Natural Sciences, Center of Excellence for Biodiversity, University of Tabriz, Tabriz, Iran

Email address of the corresponding author: mtekin@med.miami.edu
