# Supplementary material for: Whole-Exome Sequencing Efficiently Detects Rare Mutations in Autosomal Recessive Nonsyndromic Hearing Loss
Source: PLoS One. 2012 Nov 30;7(11):e50628. doi: 10.1371/journal.pone.0050628 (PMC3511533; doi:10.1371/journal.pone.0050628)
Supplement: Table S2 — Heterozygous mutations in known deafness genes in affected families and hearing individuals. HGMD: Human Gene Mutation Database. 1Minor allele frequency is from dbSNP137 database accessed on 10/17/2012. 2Internal database allele frequency does not include the family on this table with a given variant. (PDF) [file pone.0050628.s003.pdf]

Family	Gene	cDNA change	Amino acid change	NM number	SNP number	Minor Allele Frequency[1]	Internal database Allele frequency [2]	Present in HGMD	Polyphen-2 Score	SIFT	Affected Protein Domain
	Affected families										
2	*TPRN*	2083G>A	A695T	NM_001128228.2	-	-	0/1958	No	0.941	0	No
5	*CDH23*	5518C>T	R1840W	NM_022124.5	rs145951744	0.0005	1/1966	No	0.998	0.02	Cadherin repeat-like Domain
6	*PCDH15*	5717delA	N1906Tfs*18	NM_033056.3	-	-	0/2014	No	-	-	n/a
10	*USH1C*	1858C>T	R620C	NM_153676.3	rs143160805	0.000743	0/2010	No	0.961	0	No
	Hearing families										
90035	*MYO15A*	3845G>T	R1282L	NM_016239.3	-	-	0/1994	No	0.999	0.01	Myosin motor Domain
90036	*TPRN*	1256C>T	P419L	NM_001128228.2	rs149753507	0.00129	1/1886	No	0.998	0.04	No
25214	*COL11A2*	580C>T	R194C	NM_080681.2	-	-	0/1862	No	0.999	0	Laminin G Domain
25215	*MYO7A*	562C>G	Q188E	NM_001127180.1	-	-	0/1950	No	0.998	0	Myosin motor Domain
25215	*TRIOBP*	5588C>T	A1863V	NM_001039141.2	rs140528529	0.001238	5/1994	No	0.789	0	Pleckstrin homology Domain
25220	*TRIOBP*	3456C>G	D1152E	NM_001039141.2	-	-	0/1992	No	0.956	0.04	No

Article title: Whole-exome sequencing efficiently detects rare mutations in autosomal recessive nonsyndromic hearing loss

Journal name: Human Genetics

Author names: Oscar Diaz-Horta[1], Duygu Duman[2], Joseph Foster II[1], Aslı Sırmacı[1], Michael Gonzalez[1], Nejat Mahdieh[3], Nikou Fotouhi[4], Mortaza Bonyadi[4], Filiz Başak Cengiz[2], Ibis Menendez[1], Rick H. Ulloa[1], Yvonne J.K. Edwards[1], Stephan Züchner[1], Susan Blanton[1], Mustafa Tekin[1]

Affiliations:

1) John P. Hussman Institute for Human Genomics and the Dr. John T. Macdonald Department of Human Genetics, University of Miami Miller School of Medicine, Miami, USA

2) Division of Pediatric Genetics, Ankara University School of Medicine, Ankara, Turkey

3) Growth and Development Research Center, Tehran University of Medical Sciences, Tehran, Iran

4) Faculty of Natural Sciences, Center of Excellence for Biodiversity, University of Tabriz, Tabriz, Iran

Email address of the corresponding author: mtekin@med.miami.edu
